# Characteristics of cerebrospinal fluid biomarkers/ imaging findings in patient with amyloid‐negative dementia ∼second report∼

**DOI:** 10.1002/alz.088068

**Published:** 2025-01-09

**Authors:** Naoto Takenoshita, Shoya Inagawa, Ryo Yamamoto, Haruo Hanyu, Kensaku Kasuga, Takeshi Ikeuchi, Kenji Ishii, Soichiro Shimizu

**Affiliations:** ^1^ Tokyo Medical University, Tokyo Japan; ^2^ Southern TOHOKU Research Institute for Neuroscience, Tokyo Japan; ^3^ Department of Molecular Genetics, Brain Research Institute, Niigata University, Niigata, Niigata Japan; ^4^ Brain Research Institute, Niigata University, Niigata, Niigata Japan; ^5^ Tokyo Metropolitan Institute for Geriatrics and Gerontology, Tokyo Japan

## Abstract

**Background:**

In daily clinical practice, we often encounter cases with atypical symptoms and imaging findings, even among those diagnosed with Alzheimer's disease. Amyloid PET and cerebrospinal fluid examinations were performed in such non‐typical cases and young cases.

**Method:**

A total of 85 patients underwent both amyloid PET and cerebrospinal fluid examinations. The amyloid PET results were divided into two groups, amyloid‐positive group (n=43) and amyloid‐negative group (n=42) based on the visual judgment criteria of Japanese‐ADNI. Neuropsychological tests, MRI findings, cerebral blood flow SPECT findings, and cerebrospinal fluid findings were compared between the two groups.

**Result:**

CSF findings showed significant differences in Aβ42, Aβ42/40 and P‐tau between the two groups. MRI findings showed no significant differences in medial temporal lobe atrophy and cerebral white matter lesion between the two groups. Cerebral blood flow SPECT findings showed a significant difference in decreased blood flow in the right parietal lobe and bilateral precuneus between the two group.

**Conclusion:**

In dementia patients with atypical clinical symptoms/imaging findings, cerebrospinal fluid findings and decreased blood flow in the right parietal lobe and bilateral precuneus are used to investigate whether amyloid is involved in the background pathology.

